# Development and validation of self-report Mizaj identification questionnaire Based on Persian Medicine for the elders (age over 60) 

**DOI:** 10.22088/cjim.15.1.8

**Published:** 2024

**Authors:** Marjan Akhtari, Morteza Mojahedi, Narjes Gorji, Ali Bijani, Seyyed Ali Mozaffarpur, Roshanak Saghebi, Reihaneh Moeini

**Affiliations:** 1 Student Research Committee, Babol University of Medical Sciences, Babol, Iran; 2 Social Determinants of Health Research Center, Health Research Institute, Babol University of Medical Sciences, Babol, Iran

**Keywords:** Mizaj, Persian Medicine, Questionnaire, Temperament, Unani medicine, Validation assessment

## Abstract

**Background:**

Introduction: Mizaj is the basis of attention to individual differences in Persian Medicine (PM). Regarding the importance of Mizaj for health preservation and treating diseases, it is necessary to achieve a standard tool for Mizaj identification. The purpose of this study was to design a standard self-reporting Mizaj identification questionnaire for elders.

**Methods:**

In this exploratory sequential study, criteria of Mizaj identification were extracted by reviewing PM literatures and interview with PM experts and elders. The primary questionnaire was designed and its validity and reliability were assessed, using weighted Kappa statistics, Pearson correlation coefficient (PCC) assessment, receiver operating characteristic (ROC) curve and determining the specificity and sensitivity of cut-off points.

**Results:**

Among the 101 items in the primary questionnaire, 73 items had acceptable reliability. The final 20-item questionnaire was obtained after the criterion validity and PCC assessment. The sensitivity and specificity of this questionnaire were 83% and 88% for warmness, 49% and 80% for moderate in warmness-coldness, 72% and 91% for coldness, 57% and 78% for wetness, 30% and 79% for moderate in wetness-dryness, and 81% and 67% for dryness, respectively.

**Conclusion:**

The standard Mizaj identification is recommended as a supplementary diagnostic tool for clinicians and researchers in PM. Also, the people with age over 60 can use it to identify their own Mizaj and then, choose the suitable PM or Unani medicine lifestyle recommendations based on their Mizaj.

Recently, the World Health Organization (WHO) has been paying special attention to the different types of traditional medicine around the world (1, 2). Persian Medicine (PM), being one of the oldest traditional medicine literatures of the world, has been utilized during many centuries (3, 4). Mizaj (temperament) is one of the fundamental concepts and the basis of disease diagnosis and preventive and therapeutic recommendations in PM (5, 6). 

Mizaj is the basis of individualistic approach in PM which represents special physiological, physical and psychological characteristics of a person (7–9). Although every human in the world theoretically has a unique Mizaj, there are nine main groups of Mizaj classifications in PM (9). These nine groups include four simple Mizajes (warm, cold, wet and dry), four compound Mizajes (warm/wet, warm/dry, cold/wet and cold/dry) and one moderate (balanced) Mizaj (9, 10). 

According to the PM sources, there are several criteria to determine the Mizaj, which are classified into 10 groups as "Ten Mizaj Identification Criteria" (9, 11). Today’s PM experts determine the Mizaj based on their personal experiences and sometimes a single individual's Mizaj is reported differently by several experts (12). Thus, regarding the importance of Mizaj identification for health preservation and treating diseases, it is currently a concern of PM experts to achieve a standard tool that could determine the Mizaj with fewer errors. Also, the self-report questionnaire can help individuals to determine their own Mizaj to choose a lifestyle based on it and enhance their health (13, 14). Moreover, in researches that Mizaj determination is necessary; having a standard questionnaire enhances the scientific value of the research and makes it easier (15). 

Furthermore, based on PM, the age differences are one of the critical factors affecting the Mizaj, so, to determine the Mizaj correctly, the questionnaires should be designed for different age groups. There are four important age groups introduced in PM, including growth period (birth to 30y), youth period (30y-40y), midlife period (40y-60y) and elderly period (over 60y). 

So far, two standard questionnaires have been designed for the age groups of 20-60 years old (6, 14). Nevertheless, no Mizaj questionnaire has been designed yet for individuals over 60 years old. As the population aging is a global challenge (16) and the current studies in the prevention and treatment of diseases in this age range are universally conducted , designing a standard questionnaire to determine the Mizaj (as the basis for diagnosis and treatment of diseases in PM) in the elderly is one of the research priorities of the field. Therefore, this study was conducted to design a standard and reliable self-reporting Mizaj questionnaire for elders (age over 60).

## Methods

**Study design, participants, inclusion and exclusion criteria: **This study was an exploratory sequential data analysis including a qualitative phase (item generation) and a quantitative cross-sectional phase (validity assessment) (17, 18). Two groups collaborating in this study were experts in PM and the volunteered elderly.

Inclusion criteria for the PM experts were voluntary participation and having more than 5 years of clinical or research experience about Mizaj. Inclusion criteria for elders were voluntary participation, age over 60 and having the ability to read and write.

Exclusion criteria for the experts and the elders were withdrawal from voluntary participation and also any report on uncontrolled diabetes, uncontrolled hypertension, liver or kidneys dysfunction and major psychiatric diseases such as depression, mania and panic attack for the elders. 

The present research was approved by the Institutional Research Ethics Committee, Health Research Institute, Babol University of Medical Sciences, IR.MUBABOL.HRI.REC.1396.78.

**Qualitative study: Item generation:**

**Determining the Mizaj indices in elders:** In this step, the Mizaj indices were determined according to PM texts and the viewpoints of experts and interviews with elders as follows:

All of the indices related to ten Mizaj diagnostic criteria in elders were extracted by studying selected PM literatures, including Canon of Medicine (Avicenna,980-1037), Al-Maleki (Haly Abbas,10^th^cent.), Kholasat al-Hekmah (Aghili Shirazi,17^th^cent.), Zakhireh-e-Kharazmshahi (Jorjani,1042-1136), Mansuri fi Teb (Rhazes,854–925), Ketab al-Mizaj (Galen,129-210), Ketab al-Koliat (Averroes,1126-1198), Adab al-Tabib (Es-hagh Ibn Ali Rahavi,9^th^cent.), Mofarrah Al-Gholub (Hakim Arzani,17^th^-18^th^cent.) , Kholasat al-Tajarob (Baha al-Doleh,15^th^cent.) as well as articles about Mizaj which have been published in the recent years. 

A semi-structured interview with nineteen PM experts from different faculties was conducted and their experiences on Mizaj determination of elders were gathered. 

Using purposive sampling, 50 elders residing in Babol, Iran of both genders with different educational levels were selected and semi-structured interviews were conducted. They were also asked to express their opinion about their personal characteristics about the ten diagnostic criteria of Mizaj and the interviews were continued until there was no new information to add. The gathered data were imported to MAXQDA software (VERBI Software, 2018) and classified.

**Designing primary questionnaire:** Three PM experts in a team assessed all the data and designed the primary Mizaj identification questionnaire, in this step.

**Quantitative study: Validity Assessment:**

**Primary Validity Assessment:** This step includes Qualitative Face Validity and Content Validity

**Qualitative Face Validity:** Fifty eight elders of both genders with different educational levels were asked to comment on the simplicity, clarity, and semantic understanding of items of the primary designed questionnaire. Then, the mentioned PM experts made the necessary changes for the best simplicity and clarity. Some items were added and some were removed.

**Content Validity:** This step includes content validity ratio (CVR) and content validity index (CVI) determination (19, 20).

For CVR determination, options of necessity including “necessary, useful but not necessary, not necessary” were presented for each question in the primary questionnaire and then mailed to 15 volunteer PM experts asking them to choose one of the options for every item and write their comments about the items. We followed-up their answers by calling or sending e-mails for several times. For determining the CVR the following formula was used:

$\mathrm{CVR}=\frac{\mathrm{ne}-N/2}{N/2}$

(ne: number of experts who had chosen the “necessary” option, N: total number of experts)

Using Lawshe table based on the number of experts, unacceptable items were determined and removed (6).

For CVI determination, the Bausell and Waltz method was applied. 

Options of relevancy including “completely relevant, relevant, relatively relevant, not relevant” were presented for each question in the questionnaire from the first phase and mailed to 15 volunteer PM experts asking them to choose one of the four options for each item (6). We followed-up their answers by calling or sending e-mails for several times.

For CVI determination, this formula was used: dividing the number of experts, who agreed (rank three or four), by the total number of experts. Questions with a score above 0.79 were considered appropriate, between 0.70 and 0.79 needed correction, and less than 0.70 were unacceptable (21).

**Reliability Assessment:** In this step, Test-retest method and Weighted Kappa were used to evaluate the reliability (19, 22). The questionnaire extracted from previous step was given to 61 elders of both genders with different educational levels to complete and re-complete it after two weeks. Using MedCalc® statistical software (MedCalc software, 19.6) Weighted Kappa was measured for each item and values higher than 0.74 were considered excellent, between 0.60-0.74 were considered good, between 0.40– 0.60 were considered moderate and less than 0.40 were considered fair (19). Finally, a team of three PM experts decided to remove some items with Weighted Kappa less than 0.40. The questions extracted from this step, reached the next stage.

**Secondary Validity Assessment:** This step includes Gold standard determination and Criterion Validity assessment (6). 

**Gold Standard determination:** The required sample size for this stage, was considered to be 3 to 10 volunteers per item (6). 

In the first step, 300 elders participated, 206 of them were from the final step of Mizaj determination of Amirkola project (23). There was complete agreement about Mizaj of these participants among all the 5 experts which visited them according to the protocol of that study (23). Mizaj of these participants was considered as the gold standard. Among them the elders with exclusion criteria were excluded and others were contacted and asked to complete the obtained questionnaire.

**Criterion Validity** **Assessment:** Next, the elders in Gold Standard group were asked to fulfil the extracted questionnaire from the reliability step. All the data were imported to SPSS software (IBM SPSS software, 2018) and Pearson correlation coefficient (PCC) was assessed for all the items in the coldness-warmness and wetness-dryness spectrums, separately. Then a panel discussion including five experts was conducted to choose the items of the final questionnaire based on PCC of items and the relationship between Mizaj and its indices (24). Cronbach’s α coefficient was applied to assess the internal consistency (25). To determine best cut-off points, receiver operating characteristic (ROC) curve was drawn and the area under curve (AUC) was assessed. The specificity and sensitivity of the best cut-off points for both subscales of warm-cold and wet-dry were assessed in final questionnaire (26). One-Way ANOVA test was performed for two subdomains of warm-cold and dry-wet and the ability of the cut-off points to distinguish between warmness, moderate and coldness in the warm-cold subdomain and wetness, moderate and dryness in the dry-wet subdomain were evaluated. Cross tabulation was performed for warm-cold and wet-dry scales separately, and correlation coefficient was calculated to find any association between the gold standard group and questionnaire group. Chi square test was used to measure the agreement between the gold standard group and questionnaire group by calculating Kappa for warm-cold and wet-dry scales separately.

## Results

**Qualitative study: item generation:**

**Determining the indices of Mizaj in elders:** Based on PM literatures and articles about Mizaj and interviews, 35 indices of Mizaj determination were extracted and categorized into ten criteria including touch, skin colour, hair condition, muscle and fat mass, impressibility speed, physique, sleep and wakefulness, physical function, the psychic functions and quality of waste matter. We found no especial criteria for Mizaj determination in elders. Based on interviews with elders, terms in folk literature vocabulary used for these indices were gathered.

**Designing primary questionnaire:** The team of PM experts designed the primary Mizaj identification questionnaire for elders with 101 items with a five-dimensional Likert scale which included a spectrum of coldness-warmness or wetness-dryness of Mizaj identification criteria. Based on the opinions of PM experts, interviews with elders and results of two previous similar studies (6,14), the research team decided not to design any item about quality of waste matter, so these 101 items covered nine criteria of Mizaj identification.

**Quantitative study: Validity assessment:**

**Primary Validity Assessment** **:**

**Qualitative Face Validity:** For face validity assessment, the participating elders commented on 101-item primary questionnaire. Based on their comments, the research team of experts made some changes in 58 items, 5 items were removed and 6 items were added to the questionnaire. Finally, 102 items remained for the next step.

**Content Validity:** For CVR assessment, 11 PM experts assessed the 102-item questionnaire and chose one of the options of necessity as well as writing their comments about the items. Using Lawshe table and based on expert’s opinion, nine items which had a CVR less than 0.59 were removed and some changes were made in four items. Finally, 93 items remained for the next step. 

For CVI assessment, ten PM experts completed the questionnaire and based on the Bausell and Waltz method, CVI were calculated for all of the items. According to the experts’ viewpoint, 5 items were modified and 5 items which had CVI less than 0.7 were removed finally, 88 items remained. 

**Reliability Assessment:** For Reliability Assessment, the elders completed the 88-item questionnaire extracted from the previous step twice with a two-week interval. Weighted Kappa coefficient was assessed for each item. 15 items (WK<0.40) were removed and 73 items remained for the next step.

**Secondary Validity Assessment**:

**Gold Standard determination:** Two hundred and thirty elders in two categories of coldness-warmness (cold, temperate and warm) and wetness-dryness (wet, temperate and dry) were contacted and subsequently, one hundred and fifty elders completed the obtained questionnaire (table 1).

**Table 1:** Demographic data of elders whose data was used as the gold standard

	**Count (Percentage)**	**Age (SD)**
**Male**	87 (58%)	70.38 (5.94)
**Female**	63 (42%)	68.65 (5.16)
**Total**	150 (100%)	69.65 (5.67)

**Criterion Validity** **:** Here, the elders of the gold standard group completed the 73-item questionnaire extracted from the reliability step. PCC was calculated for all the items. Based on PCC and personal experience of panel experts about Mizaj identification (24), the items of final questionnaire were selected; A 15-item model for coldness-warmness and a 5-item model for wetness-dryness.

Cronbach’s α for 15-item model of coldness-warmness and 5-item model of wetness-dryness was 0.802 and 0.472, respectively.

Based on the result of ROC curve, the best cut-off points according to the AUC were selected by the team of five PM experts. Sensitivity and specificity of the cut-off points were assessed (table 2).

**Table 2:** Sensitivity and specificity of the cut-off points with their confidence interval (CI)

**Specificity**	**Sensitivity**	**Cut** **off point**	**Quality**	
) 80-95= 88% (CI	(74-91=CI) 83%	45 ≤	**Warm** **ness**	**Warmness-Coldness**
) 73-87= 80% (CI	) 32-65= (CI%49	38-44	**Moderate**	
) 86-96= (CI%91	) 58-86= 72% (CI	37 ≥	**Cold** **ness**	
) 59-76= (CI%79	) 67-95= 81% (CI	15 ≤	**Dry** **ness**	**Dryness-wetness**
) 70-89= (CI%79	) 20-40= 30% (CI	13-14	**Moderate**	
) 70-86= (CI%78	) 42-72= 57% (CI	12 ≥	**Wetness**	

According to one-way ANOVA test, the 15-item warmness- coldness subdomain can discriminate between the three groups of this subdomain (warm, temperate, cold) and cannot determine the wetness-dryness, whereas the 5-item wetness-dryness subdomain can discriminate between the three groups of this subdomain (wet, temperate and dry) and cannot determine warmness-coldness (table 3).

Cross-tabulation and Chi-Square test were performed and the result of Contingency Coefficient in warmness-coldness and wetness-dryness was 0.637 and 0.431, respectively. Kappa in warmness-coldness was 0.555 and in wetness-dryness was 0.249.

The flowchart in figure one demonstrates the study protocol and the number of items designed for the ten Mizaj identification Criteria and their decreasing trend in various steps of Validity assessment are shown in table 4. 

The final result of this study is a 20-item Mizaj identification questionnaire in PM for the elders (age over 60) with 30- 83% sensitivity and 67-91% specificity (table 5).

**Table 3:** The result of ANOVA test for 20-items Mizaj determination questionnaire in elderly

	**Warmness-coldness**				**Wetness-dryness**			
**Model**	**Mean in coldness**	**Mean in Moderate **	**Mean in warmness**	**Between Groups significancy **	**Mean in wetness**	**Mean in Moderate **	**Mean in dryness**	**Between Groups significancy**
**15-item**	35.02	40.82	49.05	**0.00**	42.59	44.06	43.25	0.616
**5-item**	14.56	13.57	13.48	0.273	11.30	13.93	16.77	**0.00**

**Table 4:** The number of items which was designed for the ten Mizaj identification criteria and their decreasing trend in various steps of validity assessment

**Mizaj Criteria**	**Number of items after each step**					
	**Item Generation**	**Face Validity**	**Content Validity**		**Reliability Test retest**	**Criterion Validity**
			**CVR**	**CVI**		
**Touch**						
**Warm - cold**	4	4	4	3	3	2
**Wet - dry**	3	2	2	2	2	1
**Muscle and fat mass**						
**Obesity and Slimming**	2	1	1	1	1	1
**fattening up quickly**	2	1	1	1	1	1
**Hair condition**						
**Amount**	3	3	3	3	3	0
**Diameter**	1	1	1	1	1	0
**hair loss**	2	1	1	0	0	0
**Model**	1	1	1	1	1	0
**A** **g** **e** ** of hair bleaching**	1	1	1	1	1	0
**Amount of white hair**	1	1	1	1	0	0
**Body Color**						
**Whole body color**	2	2	2	2	2	1
**Physique**						
**Whole body Physique**	3	3	3	3	2	2
**Palm size**	2	2	2	2	2	1
**Impressibility speed**						
**weather**	9	9	9	9	9	1
**Food Mizaj**	3	3	3	3	3	1
**Sleep and wakefulness**						
**Speed of falling speed**	2	2	2	2	2	1
**Sleep duration**	3	3	3	3	2	0
**Sleep depth**	1	1	1	1	1	0
**Physical function**						
**Speed**	5	6	6	6	4	2
**Energy**	5	6	5	5	3	1
**Voice level**	2	2	2	2	2	1
**Speech speed**	4	4	4	4	4	1
**Psychic function**						
**Desire for working**	1	2	2	2	1	0
**Hope**	3	3	2	0	0	0
**Attention**	1	1	1	1	1	0
**Grudge and pardon**	3	3	1	1	0	0
**Speed of deciding**	1	1	1	1	1	0
**Fear and ** **courage**	3	3	2	2	2	0
**Wait and hurry**	3	3	3	3	2	1
**Community relations**	6	7	6	6	3	1
**Willing to preside**	6	6	5	4	3	1
**Happiness**	3	5	4	4	3	0
**Being blunt**	3	3	3	3	3	0
**Being flexible or stubborn**	4	4	3	3	3	0
**Speed of anger**	3	2	2	2	2	0
**Quality of waste matter**	0	0	0	0	0	0
**Total**	101	102	93	88	73	20

CVR: Content validity ratio, CVI: Content validity index

**Table 5:** Self-report Mizaj identification questionnaire for the elders (age over 60)

**5**	**4**	**3**	**2**	**1**	**Questions for warm-cold**	
Warm	Somewhat warm	Neither cold nor warm	Somewhat cold	Cold	How is your body usually cold and warm?	**1**
Mostly say you are talkative	Some people say you are talkative	They say nothing special	Some people say you are incommunicative	Mostly say you are incommunicative	What do others say about the kind of talk you have?	**2**
Less	Somewhat less	Like others	Somewhat more	More	Are you dressed more or less in winter compared to your peers?	**3**
I always love it	I love it more times	I love it sometimes	I don't like it most of the time	I never like it	Would you like to take responsibility for trips or parties?	**4**
Hot	Somewhat Hot	Neither Cold nor Hot	Somewhat Cold	Cold	Do you find your social relationships cold or hot?	**5**
Eating warm Mizajs foods always lead me to feel upset	Eating warm Mizaj foods somewhat lead me to feel upset	No difference for me	Eating cold Mizaj foods somewhat lead me to feel upset	Eating cold Mizaj foods always lead me to feel upset	How do affect foods with cold Mizaj (such as yogurt and cucumbers) or foods with warm Mizaj (such as honey, spices, and peppers) on you?	**6**
Swarthy	Wheat	Normal	Almost White	White	What color does your skin have?	**7**
Large	Almost large	Moderate-Normal	Almost Small	Small	How do you describe your skeleton compared to your peers?	**8**
Hasty	Somewhat hasty	Sometimes patient and sometimes hasty	Somewhat patient	Patient	Do you identify yourself as hasty or patient?	**9**
Mostly say it's warm	Some say it is warm	They don't say anything special	Some say it is cold	Mostly say it is cold	When your peers with the gender like you, touch your hand, what do they usually say about its warmth and coldness?	**10**
Always agile	Almost agile	Neither steady nor agile	Almost steady	Always steady	How do you do your routine activity compared to your peer?	**11**
Much taller than others	Slightly taller than others	Normal	Slightly weaker than others	Much weaker than others	What is the strength of your voice?	**12**
More	Somewhat more	Like others	Somewhat less	Less	When you compare yourself to your peers, how do you see the strength of your body?	**13**
Always agile	Almost agile	normal	Almost steady	Always steady	If you do not have pain, how do you walk?	**14**
Larger	Somewhat Larger	Medium- Like Others	Somewhat Smaller	Smaller	What is the total size of your palm compared to your peers?	**15**
**5**	**4**	**3**	**2**	**1**	**Questions for wet-dry**	
Very Large	Large	Medium - Normal	Small	Very Small	How is your body compared to your peers?	**1**
Dry	Somewhat Dry	Normal	Somewhat Soft	Soft	Is your skin soft Or dry?	**2**
Slim	Slightly Slim	Neither fat nor Slim	Slightly fat	Fat	How do you feel about obesity and overweight?	**3**
slow and barely	Somewhat slow and barely	Normal	Somewhat fast & easy	Very fast and easy	How do you gain weight?	**4**
Always	Almost	Sometimes Yes Sometimes No	Rarely	Never	Do you need to take sleeping pills to sleep?	**5**

For every question, first option has one score, 2nd option has 2 scores, third option has 3 scores, 4th option has 4 scores and 5th option has 5 scores. After filling out the questionnaire, add the scores.

For Warm/cold determination, score 45 ≤ is warm, score 38-44 is mod (WC) and score 37 ≥ is cold.

For dry/wet determination, score 15 ≤ is dry, score 13-14 is mod (WD) and score 12 ≥ is wet.

**Figure 1:**
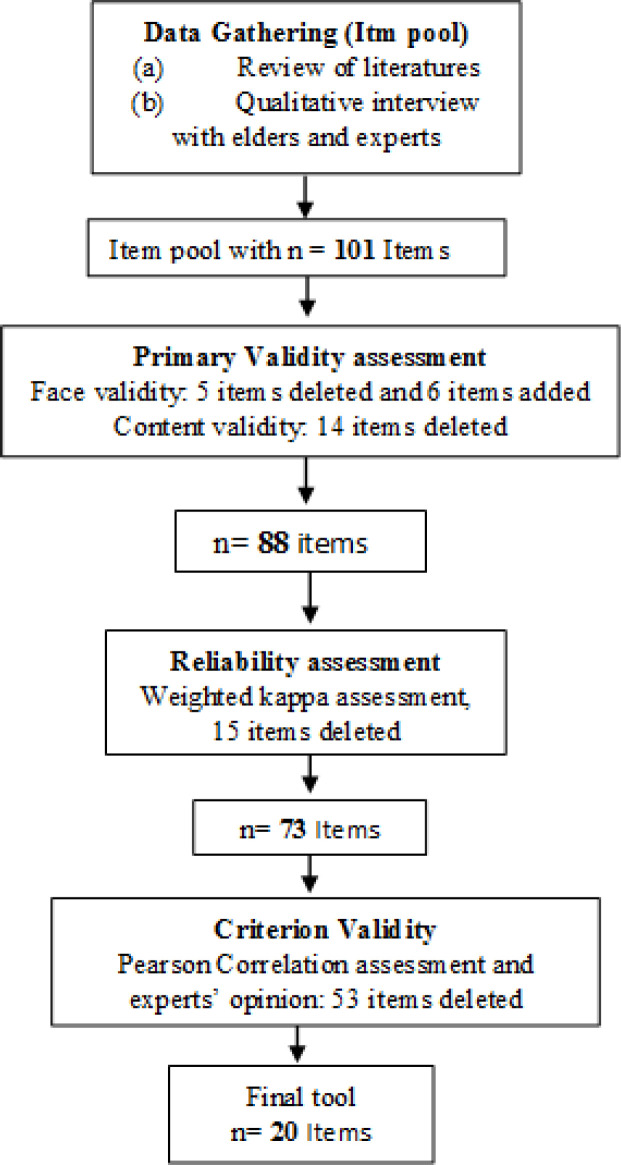
Flowchart of study protocol

## Discussion

In this study, a unique self-report Mizaj questionnaire was developed and validated with a 15-item model for the coldness-warmness differentiation and a 5-item model for wetness-dryness differentiation for elders with age over 60.

The determination of Mizaj is one of the essential steps for health preservation and diagnosis and treatment in PM, so much as all the prescriptions of health in PM are categorized based on the Mizaj groups (27). Over the past few decades, personalized medicine has become a new paradigm of medical attention and aaccording to the idea, individual differences affect the presentation and severity of the disease along with the scale of response to treatment (28). With regards to the PM approach as a personalized medicine, the issue of Mizaj can have a significant impact on the advances in personalized medical goals. 

So far, two standard Mizaj questionnaires have been developed based on age groups, which can be compared with the current study in different aspects. Mojahedi et al. (2014) designed and validated a ten-item questionnaire for the age 20-40y (MMQ). Despite the simplicity and utility of MMQ, their study had some limitations including the lack of some criteria in the questionnaire, the low number of samples and low sensitivity and specificity for wet/dry dimension (14). 

SalmanNejad et al. (2017) developed a 20-item Mizaj questionnaire for people aged 20-60y (SMQ). This questionnaire was more sensitive than MMQ, whereas some criteria were still missing from the questionnaire (6). 

The current study contains a larger sample size (150 volunteers) compared to MMQ (n=52) and smaller sample size compared to the SMQ study (n=224). In this investigation, at the Gold Standard setting stage, five constant PM specialists visited each individual who were reported four to eight in the MMQ study and were three in SMQ study. In this study, the initial questionnaire was developed with 101 items, which was noticeably higher than of MMQ study (52 items) and lower than the SMQ study (119 items). Moreover, no question was designed about the quality of waste matter criteria, whereas in the initial questionnaires of Mojahedi and SalmanNejad, there were questions about all the ten criteria of Mizaj identification.

In this study, more items were entered into the criterion validity phase than both the MMQ and SMQ studies and ultimately, a 20-item questionnaire was achieved that included more questions than the MMQ (10-item) and was similar to the SMQ in the number of items and coverage of the nine criteria of Mizaj, whereas the MMQ covered only six criteria. 

Considering the final results, the sensitivity of the cut-off points in warmness, coldness, dryness and wetness is higher than MMQ, whereas their specificity is very close. Furthermore, the sensitivity of the cut-off points in coldness, moderate (Wetness-Coldness) and wetness and moderate (Wetness-Dryness) of the present scale is lower than SMQ, but the specificity of coldness, warmness, moderate (Wetness-Coldness), wetness and moderate (Wetness-Dryness) is higher than SMQ. Thus, the researchers recommend this questionnaire and MMQ for diagnostic purposes of Mizaj researches in PM and the SMQ for the screening Mizaj studies.

Roshandel et al. carried out a study to design and standardize a Mizaj questionnaire in 2015. The disadvantage of their study was their obese and not community-based samples. Furthermore, the face validity was not evaluated in a complete and correct way, and it seems that content validity and some validation steps were not correctly implemented. Moreover, they could not assess the Criterion Validity due to lack of Gold Standard; so it is not comparable to the current study (10).

Roshandel et al. also designed and developed a software to determine Mizaj based on an algorithm of facial dimensions and appearance in another study. In their study, results of software analysis were compared by 3 experts’ opinion and the results of a 26-item questionnaire (containing questions about color of skin, color of head hair, facial structure, forehead size, eyes to face ratio, iris color, color and condition of the sclera and other characteristics of the head and shoulders). Finally, almost complete agreement between software and expert’s opinion and moderate agreement between software and questionnaire were obtained. The stages of questionnaire designing have not been stated in their study, and aim of the study was the determination of Mizaj according to face indices, so, its not comparable with our study (29).

Other standard studies have been conducted on designing and developing diagnostic tools for Mizaj specific to organs Mizaj, such as Fattahi Masoom et al.’s study which resulted in a 35-item questionnaire and a 12-item checklist for distinguishing brain Mizaj (30).

One of the special advantages of our study is its Gold Standard setting method. Here, the recording technique was employed that is an innovative method due to a large number of volunteers (n=300) and the inability to coordinate the attendance of the five specialists and volunteers (23). Although its accuracy is somewhat less than the face to face visit, this method can be utilized in future researches and those situations that the presence of a volunteer and several specialists is not possible.

Although in the present study, a good reliability and validity were obtained for the questionnaire, due to the possibility of low literacy or the difficulty of reading the questionnaire in the elderly, the use of a checklist along with the questionnaire probably lead to more reliable results. The important point of this questionnaire was to pay attention to the age characteristics and limitations such as consumption of sleeping pills and musculoskeletal pain in the design of the questions.

This study has some limitations including the small number of samples in the Gold Standard group (considering the number of items). The reason for this difficulty was the low collaboration of this age group. The other limitation is the low sensitivity, specificity, Cronbach's alpha and Kappa in the wet-dry group and also the non-attendance Mizaj identification of volunteers by specialists in the Gold-standard setting phase. Moreover, since the sample size in the two steps of this study was chosen from the people in Babol, it is recommended to evaluate the validity and reliability of the study in a multicultural city like Tehran for more accuracy.

The final outcome of this study is a 20-item Mizaj identification questionnaire for elders with age over 60, which passed the steps of validity and reliability appraisement of a standard diagnostic tool in medical research. It is the first standard self-reporting Mizaj identification questionnaire for elders and the third standard questionnaire after MMQ and SMQ. Therapists and researchers can use it as a supplementary scale for Mizaj identification in elders, also the people with age over 60 can use it to identify their own Mizaj and then, choose the suitable PM or Unani medicine lifestyle recommendations based on their Mizaj.
